# Osteoporosis in Polish Older Women: Risk Factors and Osteoporotic Fractures: A Cross–Sectional Study

**DOI:** 10.3390/ijerph17103725

**Published:** 2020-05-25

**Authors:** Agnieszka Nawrat-Szołtysik, Zuzanna Miodońska, Ryszard Zarzeczny, Izabela Zając-Gawlak, Józef Opara, Alicja Grzesińska, Beata Matyja, Anna Polak

**Affiliations:** 1 Department of Physiotherapy in Internal Medicine, Faculty of Physiotherapy, The Jerzy Kukuczka Academy of Physical Education in Katowice, 40-065 Katowice, Poland; a.polak@awf.katowice.pl; 2 Department of Informatics and Medical Devices, Faculty of Biomedical Engineering, Silesian University of Technology, 41-800 Zabrze, Poland; zuzanna.miodonska@polsl.pl; 3 Department of Physiology and Sports Medicine, Faculty of Physical Education Chair of Biomedical Sciences, Józef Piłsudski University of Physical Education, 00-968 Warsaw, Poland; ryszard.zarzeczny@awf.edu.pl; 4 Department of Theory and Methodology of Physical Education, Faculty of Physical Education, The Jerzy Kukuczka Academy of Physical Education in Katowice, 40-065 Katowice, Poland; izazaja@wp.pl; 5 Department of Physiotherapy in Movement System and Development Age Diseases, Faculty of Physiotherapy, The Jerzy Kukuczka Academy of Physical Education in Katowice, 40-065 Katowice, Poland; j.opara@awf.katowice.pl; 6 Rehabilitation Clinic Technomex, 44-141 Gliwice, Poland; alicjakucharska05@gmail.com; 7 Center Saint Elizabeth, 41-700 Ruda Śląska, Poland; beatamatyja@interia.pl

**Keywords:** osteoporosis, risk factors, osteoporotic fractures

## Abstract

*Background:* Osteoporosis is a skeletal disease. It is still not known which of the risk factors have the greatest impact on osteoporosis development. The study aimed to determine how the selected osteoporosis risk factors contribute to the development of the disease and to assess the risk of osteoporotic fractures in older women. *Methods:* A cohort of 99 older females was divided into two groups (with and without osteoporosis). The risk of osteoporosis was determined using assessment forms and bone densitometry data subjected to logistic regression. The risk of osteoporotic fractures was assessed by the FRAX tool (FRAX, Center for Metabolic Bone Diseases, University of Sheffield, UK). *Results:* The logistic regression analysis showed that the highest risk of developing osteoporosis associated with lifestyle, mainly cigarette smoking (odds ratio: OR = 2.12), past gynecological operations (OR = 1.46), corticosteroid therapies (OR = 1.38). More than half of participants were at a medium risk of femoral neck fractures (over 90% in the osteoporotic group). *Conclusion:* Most of the Polish women living in care facilities are at medium risk of low-energy fractures. Smoking appeared to have the strongest effect on osteoporosis among analyzed risk factors. The results may contribute to the creation of more appropriate prevention strategies.

## 1. Introduction

Osteoporosis reduces bone strength and, consequently, increases the risk of bone fracture from falls. Low peak body mass (PBM) and accelerated deossification are indicated to raise the probability of osteoporotic fractures, while high and gradually decreasing PBM make them less likely. In people with osteoporosis, even minor traumas can lead to fractures of the vertebrae, the proximal end of the femur, the proximal end of the humerus, ribs, the pelvis, and the proximal end of the tibia [1]. According to United States’ data, around 2 million osteoporotic fractures, of which 300,000 are femoral neck fractures, are managed in the country each year. The international epidemiological data on proximal femur fractures are alarming. It is forecasted that their number will continue to rise, reaching 4 million in 2025, and more than 6 million in 2050. For the sake of comparison, in 1990, only 1.6 million proximal femur fractures were recorded. This trend has been very aptly termed ‘an epidemic of fractures’ [2]. According to Lopez-Lopez et al., osteoporotic fractures are also related to foot problems, which are a common problem [3]. Studies show that foot disorders occur in over 70% of older patients [4]. Osteoporotic fractures are more common in populations living in the north of Europe, especially in Scandinavia, than in Mediterranean countries, and in large cities than in rural areas [5].

The risk factors for osteoporosis have been divided into three categories. The so-called non-modifiable (uncontrollable) factors include old age, female gender, genetic predispositions, and race (Caucasian and Asian). The modifiable factors (partly controllable) are leanness, low body mass, estrogen deficiency, early menopause (before the age of 45), hyperthyroidism, type 1 diabetes, rheumatic diseases (including ankylosing arthritis and ankylosing spondylitis), received treatment with corticosteroid therapies or other drugs against chronic diseases, anti-leptic drugs, stomach-protecting drugs containing aluminum, tranquilizers, heparin, oral anticoagulants, anti-tuberculosis drugs, chemotherapeutic agents, tetracyclines, diuretics. The third category of osteoporosis risk factors (fully-controllable factors) include lifestyle, and the most common are cigarette smoking, excessive coffee and alcohol consumption, sedentary lifestyle, calcium-poor diet, and insufficient dietary intake of vitamin D and proteins [6,7].

How each of the risk factors contributes to the development of osteoporosis is yet to be established, but the concomitance of several of them is reported to carry a higher risk of developing the disease than one factor. The risk even increases when the factors represent different categories [8]. A survey of people aged 60+ on the Polish population conducted by Jędrzejczak et al. showed that older people have limited knowledge of the causes of osteoporosis [9]. According to the survey participants, osteoporosis development was mainly induced by the use of stimulants, hard physical work in the past, and old age. At the low end of the ranking were gender, genetic and family predispositions, and endocrine disorders. Having surveyed 100 women aged 50+ (mean age 55.5 years), Podbielska et al. reported that 8 of them were very well aware of the preventive and therapeutic role of exercise in osteoporosis, 27 respondents were aware of it, but 42 and 14 respondents knew little or very little about the relationship between exercise and osteoporosis [8]. Podbielska et al. also found an association between respondents’ knowledge and their place residence. Those living in urban areas showed significantly better knowledge of the anti-osteoporotic effect of physical activity than rural respondents (*p* ˂ 0.05) [10].

Assessment of patients’ risk factors for osteoporosis and of their susceptibility to osteoporotic fractures is internationally recognized as a vital element of diagnosis because, as well as making the treatment of individual patients more effective, it also provides guiding information for the development of optimal of prevention strategies.

Given the above context, this study aimed to extend the knowledge about some risk factors for osteoporosis and low-energy fractures in older women in the Polish population. The research was conducted to verify two hypotheses:

*1. Some osteoporosis risk factors have a greater impact on the disease development than others.*

*2. The risk of osteoporotic fractures is high in older women living in residential facilities.*

## 2. Materials and Methods 

The study was carried out with the female residents of 4 care facilities in the years 2009–2010. The inclusion criteria were formulated as follows: (1) age 65 or older, (2) permanent resident of a care facility, (3) knowledgeably consenting to participate in the study. The exclusion criteria were: (1) women younger than 65 years, (2) dementia, Alzheimer’s disease, reduced cognitive functioning, incapability of understanding the study protocol, or communicating with other people, (3) nationality other than Polish. 

Of 131 women aged 65–98 years who volunteered to participate in it, 32 were found ineligible after screening with inclusion and exclusion criteria. All of the remaining 99 women provided voluntary informed written consent to participate in the study. The study protocol was approved by the Ethics Commission at the Academy of Physical Education in Katowice (decision No.7/2009), as it followed all the legal and ethical standards required by adequate national and European organizations.

The mean age of participants was 81 years (Min. 65 Max. 98; SD 7.63), mean BMI (body mass index) 26.9 (Min. 16.7 Max. 41.7; SD 4.79), and mean bone density (T–Score) as determined by densitometry was 3.07 (Min. –5.4 Max. 0.0; SD 1.17). Before the study, participants spent an average of 4.7 years in the facilities.

The data collection protocol was designed as follows. In the first phase, the introductory interview and measurements were performed. The collected characteristics included age, duration of stay in a care facility, BMI, and data concerning osteoporosis risk factors. Participants’ data records included information about their diseases (diabetes, ankylosing arthritis, hyperthyroidism), past dietary habits (considering dairy), the use of stimulants and medications (hormone replacement therapy, corticosteroids), gynecological operations, the number of births, age of menopause, and level of physical activity in the past. The specific questions were read aloud to participants who ticked the appropriate boxes on the survey forms.

The second part of the data collection protocol was bone mass density examination. According to the American College of Radiology, forearm bone density assessment can be used for osteoporosis diagnosis in patients over 50 years of age when central densitometry is not available [1]. As our study was such a case, we decided to perform peripheral densitometry in care facilities where the participants lived. Forearm bone density was assessed in participants using a peripheral DXA scanner (PIXI, GE Medical Systems Lunar, Madison, WI, USA). USA Forearm Reference Population data were used as a reference for T-Score computation.

The 10-year risk of participants sustaining a proximal femur fracture or any other main osteoporotic fracture (clinical vertebral fracture, forearm fracture, fracture of the distal femur or the distal humerus, femoral neck fracture) was assessed by the FRAX tool available at www.shef.ac.uk/FRAX. This calculator is based on the prospective assessment of fracture risk in 230,486 patients from Europe, North America, Asia, and Australia. FRAX was approved by WHO and 35 countries, including Poland [11]. The following participants’ characteristics were used for calculations: bone mass density (BMD), gender, age, maternal history of hip fractures, long-term corticosteroid therapies, ankylosing arthritis, smoking, consumption of alcohol, and diseases leading to secondary osteoporosis [11].

FRAX scores are presented as percentage values between 0% and 100%. In Poland, they are usually divided into three intervals – <10%, 10–20%, and >20%, which indicate a low, moderate, and high risk of osteoporotic fractures, respectively [12,13].

The importance of particular risk factors in the development of osteoporosis was assessed with logistic regression analysis, which yielded a discriminant function and odds ratios (OR) for all factors. The power of the discriminant function was subsequently assessed through a classification experiment performed on the initial dataset. Obtaining a statistically significant classification power of the discriminant function was treated as a criterion of sample size adequacy.

The 10-year risk of participants sustaining a femoral neck fracture or the main types of osteoporotic fractures is presented as descriptive statistics. The level of statistical significance was been set at *p* < 0.05. All calculations were performed in STATISTICA v.10 by StatSoft (StatSoft, Hamburg, Germany).

## 3. Results

The assessment of particular osteoporosis risk factors was carried out separately for osteoporotic women (T–Score < –2.5; N = 74) and non-osteoporotic women (T–Score > –2.5; N = 25; 16 with osteopenia and 9 with a normal T–Score). The basic characteristics of both groups of participants are shown in Table 1. Table 2 shows particular risk factors for osteoporosis and the numbers and percentages of participants affected by them.

The logistic regression analysis showed that the highest risk of developing osteoporosis was associated with cigarette smoking (OR of 2.12), gynecological operations (1.46), corticosteroid therapies (1.38), maternal history of hip fractures (1.29), low intake of dairy products (1.19), sedentary lifestyle (1.17), and hormone replacement therapies (HRT) (1.09) (Table 3). At the same time, none of the ORs was statistically significant, which means that none of the factors individually had a significant effect on the development of osteoporosis.

The discriminant function results were used to classify participants as osteoporotic or non-osteoporotic. The accuracy of this classification (i.e., the percent match between its outcomes and the actual health status of participants) was 75%. The odds ratio (OR) for participants being correctly classified was statistically significant and was equal to 4.37, with the confidence interval ranging from 1.073 to 17.84 (Table 4).

The FRAX showed that the mean probability of participants sustaining all main types of osteoporotic fractures was 29% (the minimum and maximum probabilities were 5.9% and 69%, respectively) and the mean risk of a humeral fracture was 17% (0.7% and 65%) (Table 5). The mean risk of main osteoporotic fractures and femoral neck fractures in the groups was 31% vs. 24.55% and 19.5% vs. 11.38%, respectively.

The analysis of FRAX scores also showed that in the osteoporotic group, as many as 91.9% of participants were at a high or medium risk of a femoral neck fracture, compared with only 76% in the non-osteoporotic group. When the entire group is taken into consideration, the risk of femoral neck fractures was high for 32.3% of participants, average for 55.6%, and low for 12.1% (Table 6).

## 4. Discussion

Old people are at higher risk of developing osteoporosis, which doubles every decade after the age of 65 years [14]. After examining bone mineral density in 81 women aged 64–75 years, Skrzek et al. found that 21% of them had osteoporosis and 53% osteopenia [15]. The rates correspond to those published by the World Health Organisation, according to which around 54% of Caucasian postmenopausal women have osteopenia and 30% osteoporosis. For women aged 80 and older, the respective rates are 27% and 70% [16]. In our study involving 99 women at a mean age of 81 years, 74.8% suffered from osteoporosis and 16.2% from osteopenia. Bone mineral density of the other 9.1% of women was normative. 

Low intake of milk and dairy calcium is also a risk factor for osteoporosis confirmed by our study. A deficiency of dietary calcium, also present in green vegetables, leguminous plants, and fish, is associated with increased secretion of parathyroid hormones and bone calcium resorption. Although the modified healthy eating pyramid recommends that older people should have three portions of milk and dairy products a day [17], many research reports indicate that dairy products and calcium-containing foods account for a small portion of their diet. A Poland-wide survey found that 86.8% of female adult respondents were deficient in calcium [18]. In the study by Skop-Lewandowska et al. involving 66 women and 62 men aged 73.2 ± 6.9, an average respondent would consume calcium-rich foods (yogurt, kefir, buttermilk, cottage cheese) only 2–3 times a week, with the weekly intake of dietary calcium accounting for only 26% of the recommended amount [19]. Green vegetables and leguminous plants were consumed once a week and fish once a week, on average. 

Our study confirmed that factors such as a sedentary lifestyle, maternal history of hip fractures, early menopause (before the age of 45), more than three births, gynecological operations, corticosteroid therapies, and HRTs could increase the risk of osteoporosis. Similar results were reported by Bączyk, Chuchracki, and Klejewski, who examined 85 women at a mean age of 59.9 ± 5.20 years [20]. A multi-factor ANOVA revealed an association between participants’ BMD and their age (*p* = 0.009), BMI (*p* = 0.002), maternal history of hip fractures (*p* = 0.005), bisphosphonate therapy (*p* = 0.001), transcutaneous HRT (*p* = 0.03), and the level of physical activity (*p* = 0.04). 

The role of physical activity in preventing the development of osteoporosis is attributed to its ability to increase bone strength and BMD, and thus, the mechanical properties of the skeletal system, and to normalize abnormal bone remodeling [21]. Epidemiological studies have shown an increased risk of femoral neck fractures in women with a maternal history of such fractures [22]. Other major risk factors for osteoporosis are early loss of the ovarian function (spontaneous or caused by resection, chemotherapy, or radiotherapy), resulting in estrogen deficiency before the age of 45 years and corticosteroid therapies [23]. The latter induces the development of vitamin D resistance by reducing intestinal calcium absorption and increasing calciuria, which may lead to secondary hyperparathyroidism associated with bone mass loss. In patients on corticosteroid therapies, bone histology results vary depending on the dose and length of treatment. Larger doses of corticosteroids that most patients receive in the early treatment phase are associated with strong bone resorption, but during long-term therapies, bone formation is mainly observed [2]. According to many research reports, the HRT effectively reduces the risk of vertebrae and forearm bone fractures, but it is contraindicated for women without strong menopausal symptoms and should be discontinued after the age of 60 years [24].

Our study confirmed that of all analyzed factors smoking cigarettes is associated with the highest risk of osteoporosis. The finding is consistent with the results reported by Hannan et al., who monitored mineral bone density in 800 men and women at a mean age of 74 ± 4.5 years [25]. The authors, too, pointed to cigarettes as a key factor in BMD reduction. Other risk factors they indicated were low BMI, unreplaced estrogen deficiency during menopause, and excessive alcohol consumption. They also reported that a greater decrease in trochanteric bone density was observed in male smokers compared with male non-smokers. 

Epidemiological studies also showed that smokers are at higher risk of developing osteoporosis. Smoking is reported to correlate positively with vertebrae, forearm bones, and hip fractures, but the exact nature of this relationship is yet to be determined. It is presumed that inhaling cadmium from tobacco smoke impairs calcium uptake, inhibits osteoblast activity, and increases oxidative stress in bone cells. It is also indicated that nicotine, too, has a detrimental effect on bone tissue. According to the existing evidence, it reduces bone mass and estrogen concentration by increasing the cortisol level, which explains its strong suppressing effect on estrogen metabolism in women [26].

The most severe problem associated with osteoporosis is bone fractures. The precise identification of people at risk of fracture is a major diagnostic challenge [27,28]. In recent years, a number of tools enabling the assessment of fracture risk have been created, e.g., the FRAX calculator recommended by the World Health Organisation and the International Osteoporosis Foundation (IOF) [26]. In our study, FRAX scores indicated that participants were at high risk of osteoporotic fractures, mostly femoral neck fractures. Experimental trials have shown that the strength of an old femur is half that of a young femur and that it can be damaged by one-third of the energy required to break a healthy bone. The probability of a 50-year old Caucasian woman sustaining an osteoporotic fracture is estimated at 40% (17.5% for a humeral neck fracture and 16% for forearm bones and vertebral fractures). It is reported that the risk of osteoporotic fracture increases after 50 years of age for every eighth man and every third woman [29]. This, and the fact that low-energy osteoporotic fractures are a frequent cause of death at old age, necessitates the development of adequate preventive measures. Górecki and Chmielewski estimated that 7% of people aged 50–74 years die within twelve months following an osteoporotic fracture, 18% of those aged 75–84 years, and 27% in the age group 75+ [30]. The highest mortality is caused by proximal femur fractures that incapacitate patients and make them dependent on other people. Marcinowska-Suchowierska et al. reported that 20% of patients with osteoporotic proximal femur fractures she studied died within the first six months from injury, and 50% after a year [31]. Most survivors are permanently disabled and suffer from a lower quality of life. The fact that many of them cannot perform daily activities unaided increases the total medical costs of managing osteoporosis. Marcinowska et al. studied 17 women and 6 men (aged 72–92 years, mean age 77.4 years) with proximal femoral neck fractures, finding that their health deteriorated after the injury so much that they had to live with a relative [32].

The presented research was aimed at determining which risk factors have the greatest impact on osteoporosis development. The results may contribute to the creation of more appropriate osteoporosis prevention strategies. These strategies should include education on, *i.a.*, adequate nutrition and physical activity, but also, according to the results, on the crucial role of avoiding smoking. Obtained FRAX scores confirm that osteoporotic fractures constitute a serious risk for older women. According to our knowledge, no works showing this kind of analysis for Polish older women have been reported before. It should also be noted that participants of the studied group were at a mean age of 81. This fact can be considered a specific strength of the research, as most of the literature on osteoporosis risk factors and osteoporotic fractures describe only younger subjects. 

Some limitations of the study should be acknowledged, as well. The classification of the participants was performed based on a peripheral DXA scan, as central densitometry was unavailable for the studied group. The research was conducted considering only women living in care facilities. The analysis was mostly retrospective (considering risk factors from the past), but still, it is difficult to determine whether this fact could have affected the results. These limitations can be treated as directions for future research. It is planned to extend the research to a larger number of subjects, including men. We are also considering conducting similar analysis with a group of women living on their own or with their families to verify whether the fact of living in a care facility has any impact on the findings. The research could also be supplemented with interviews on osteoporosis risk factor awareness among older people. Such results could then be employed in planning more successful information campaigns.

## 5. Conclusions

Of all analyzed factors, smoking carries the highest risk of inducing osteoporosis. Other main risk factors include gynecological operations, corticosteroid therapy, maternal history of hip fractures, low intake of dairy products, and a sedentary lifestyle. FRAX scores showed that women participating in the study were mostly at medium risk of low-energy fractures, mostly femoral neck fractures.

## Figures and Tables

**Table 1:** Participants’ characteristics.

	All Participants (N = 99)				Non-osteoporotic Participants (N = 25)				Osteoporotic Participants (N = 74)			
	Mean	St. dev.	Min	Max	Mean	St. dev.	Min	Max	Mean	St. dev.	Min	Max
**Age**	80.58	7.63	65	98	76.28	7.62	65	95	82.03	7.11	65	98
**BMI**	27.32	4.79	16.7	41.5	30.71	5.02	21.9	41.5	26.18	4.15	16.7	37.4
**T–score**	–3.05	1.17	–5.40	0.00	–1.44	0.68	–2.40	0.00	–3.59	0.72	–5.40	–2.50

BMI—body mass index.

**Table 2:** The numbers and percentages of participants affected by particular risk factors for osteoporosis.

	All Participants (N = 99)				Non-osteoporotic Participants (N = 25)				Osteoporotic Participants (N = 74)			
Risk factors	**YES**		**NO**		**YES**		**NO**		**YES**		**NO**	
	**No.**	**%**	**No.**	**%**	**No.**	**%**	**No.**	**%**	**No.**	**%**	**No.**	**%**
Maternal history of hip fractures	19	19.2	80	80.8	5	20	20	80	14	18.9	60	81.1
Diabetes	28	28.3	71	71.7	11	44	14	56	17	23.0	57	77
Rheumatoid arthritis	23	23.2	76	76.8	9	36	16	64	14	18.9	60	81.1
Hyperthyroidism	14	14.1	85	85.9	4	16	21	84	10	13.5	64	86.5
HRT	7	7.1	92	92.9	2	8	23	92	5	6.8	69	93.2
>3 births	12	12.1	87	87.9	2	8	23	92	10	13.5	64	86.5
Gynaecological operations	31	31.3	68	68.7	7	28	18	72	24	32.4	50	67.6
Age at menopause <45	16	16.2	83	83.8	4	16	21	84	12	16.2	62	83.8
Corticosteroids	18	18.2	81	81.8	3	12	22	88	15	20.3	59	79.7
Low intake of dairy products	31	31.3	68	68.7	7	28	18	72	24	32.4	50	67.6
Cigarettes	20	20.2	79	79.8	4	16	21	84	16	21.6	58	78.4
Alcohol	13	13.1	86	86.9	6	24	19	76	7	9.5	67	90.5
>3 cups of coffee per day	7	7.1	92	92.9	2	8	23	92	5	6.8	69	93.2
Sedentary lifestyle	56	56.6	43	43.4	13	52	12	48	43	58.1	31	41

HRT—hormone replacement therapies.

**Table 3:** Odds ratios for osteoporosis risk factors calculated from logistic regression analysis.

Risk Factors	OR	−95% CI	+95% CI
Maternal history of hip fractures	1.29	0.26	6.31
Diabetes	0.37	0.11	1.14
Rheumatoid arthritis	0.36	0.10	1.23
Hyperthyroidism	0.85	0.17	4.10
HRT	1.09	0.10	11.05
>3 births	1.02	0.66	1.57
Gynaecological operations	1.46	0.44	4.84
Age at menopause <45 years	1.02	0.93	1.12
Corticosteroids	1.38	0.29	6.46
Dairy products	1.19	0.60	2.36
Cigarettes	2.12	0.49	9.06
Alcohol	0.33	0.06	1.75
>3 cups of coffee per day	0.92	0.42	2.01
Sedentary lifestyle	1.17	0.07	17.62

HRT—hormone replacement therapies.

**Table 4:** Participants’ groups according to the results of discriminant function.

Actual Health Status	Classification Outcome		
	Healthy	Affected	Total
Non-osteoporotic	5	20	25
Osteoporotic	4	70	74
Total	9	90	99
OR = 4.3750			
alpha = 0.05			
−CI (95%) = 1.073			
+CI (95%) = 17.840			

OR—odds ratio; CI—confidence interval.

**Table 5:** The ten-year fracture risk, according to the FRAX (%).

Type of fracture	All Participants (N = 99)				Non-osteoporotic Participants (N = 25)				Osteoporotic Participants (N = 74)			
	Mean	St. dev.	Min	Max	Mean	St. dev	Min	Max	Mean	St. dev.	Min	Max
Main osteoporotic fractures	29.39	13.42	5.90	69.00	24.55	11.84	5.90	48.00	31.03	13.60	9.00	69.00
Femoral neck fracture	17.49	13.54	0.70	65.00	11.38	8.276	0.70	35.00	19.56	14.37	1.70	65.00

**Table 6:** The ten-year femoral neck fracture risk, according to the FRAX.

Risk of Femoral Neck Fracture	All Participants (N = 99)		Non-osteoporotic Participants (N = 25)		Osteoporotic Participants (N = 74)	
	No.	%	No.	%	No.	%
Low	12	12.12	6	24.00	6	8.11
Medium	55	55.56	12	48.00	43	58.11
High	32	32.32	7	28.00	25	33.78
